# High-frequency oscillatory ventilation guided by transpulmonary pressure in acute respiratory syndrome: an experimental study in pigs

**DOI:** 10.1186/s13054-018-2028-7

**Published:** 2018-05-09

**Authors:** Philipp Klapsing, Onnen Moerer, Christoph Wende, Peter Herrmann, Michael Quintel, Annalen Bleckmann, Jan Florian Heuer

**Affiliations:** 1 Department of Anesthesiology, Intensive Care Medicine, Emergency Medicine and Pain Management, University Medical Center Göttingen, Göttingen, Germany; 2 Department of Medical Statistics, University Medical Center Göttingen, Göttingen, Germany; 3 Department Anesthesiology, Intensive Care Medicine, Emergency Medicine and Pain Management, Augusta-Kliniken Bochum-Mitte, Bochum, Germany

**Keywords:** Volume controlled ventilation, HFOV, Transpulmonary pressure, Aerated lung tissue, Oxygenation, Hemodynamics

## Abstract

**Background:**

Recent clinical studies have not shown an overall benefit of high-frequency oscillatory ventilation (HFOV), possibly due to injurious or non-individualized HFOV settings. We compared conventional HFOV (HFOV_con_) settings with HFOV settings based on mean transpulmonary pressures (P_Lmean_) in an animal model of experimental acute respiratory distress syndrome (ARDS).

**Methods:**

ARDS was induced in eight pigs by intrabronchial installation of hydrochloric acid (0.1 N, pH 1.1; 2.5 ml/kg body weight). The animals were initially ventilated in volume-controlled mode with low tidal volumes (6 ml kg^− 1^) at three positive end-expiratory pressure (PEEP) levels (5, 10, 20 cmH_2_O) followed by HFOV_con_ and then HFOV P_Lmean_ each at PEEP 10 and 20.

The continuous distending pressure (CDP) during HFOV_con_ was set at mean airway pressure plus 5 cmH_2_O. For HFOV P_Lmean_ it was set at mean P_L_ plus 5 cmH_2_O. Baseline measurements were obtained before and after induction of ARDS under volume controlled ventilation with PEEP 5. The same measurements and computer tomography of the thorax were then performed under all ventilatory regimens at PEEP 10 and 20.

**Results:**

Cardiac output, stroke volume, mean arterial pressure and intrathoracic blood volume index were significantly higher during HFOV P_Lmean_ than during HFOV_con_ at PEEP 20. Lung density, total lung volume, and normally and poorly aerated lung areas were significantly greater during HFOV_con_, while there was less over-aerated lung tissue in HFOV P_Lmean_. The groups did not differ in oxygenation or extravascular lung water index.

**Conclusion:**

HFOV P_Lmean_ is associated with less hemodynamic compromise and less pulmonary overdistension than HFOV_con_. Despite the increase in non-ventilated lung areas, oxygenation improved with both regimens. An individualized approach with HFOV settings based on transpulmonary pressure could be a useful ventilatory strategy in patients with ARDS. Providing alveolar stabilization with HFOV while avoiding harmful distending pressures and pulmonary overdistension might be a key in the context of ventilator-induced lung injury.

## Background

Studies have shown that volume-controlled ventilation (VCV) with small tidal volumes, adequate positive end-expiratory pressure (PEEP) and low driving pressures (<15cmH_2_0) can improve oxygenation and reduce pulmonary morbidity in patients with acute respiratory distress syndrome (ARDS) [1, 2].

High frequency oscillatory ventilation (HFOV) is another approach to lung-protective ventilation, since it employs very low tidal volumes and very small changes in delta pressure [3] applied with higher continuous distending pressure (CDP). Several earlier studies have demonstrated the efficacy of HFOV in patients with ARDS in whom VCV has failed [4–6]. There is also evidence that outcome is improved when HFOV is initiated at an early stage [7, 8]. However, two recent studies showed either no benefit or even a higher mortality rate with HFOV compared to conventional ventilation [9, 10]. One possible explanation is that inappropriate HFOV ventilator settings had cancelled out the positive effects of HFOV.

Until now, HFOV ventilator settings have been guided by the mean airway pressure (Paw_mean_), and the CDP has been set at Paw_mean_ plus 5 cm H_2_0 in almost all studies [4, 7, 11]. This approach is more than questionable, because the Paw is not a valid surrogate for transpulmonary pressure (P_L_). Since only a positive end-exspiratory P_L_ can prevent cyclic opening and closing and overdistension of the alveolae, P_L_ has to be > 0 in order to prevent alveolar collapse.

The potential solution thus lies in choosing HFOV settings based on a more exact approach to the distending pressure applied to the lung. Talmor et al. showed that oxygenation and pulmonary compliance improves when PEEP is adjusted according to esophageal pressure (Pes) [12]. In an earlier study we found that we were able to reduce CDP when it was adjusted according to Pes [13]. It is therefore reasonable to hypothesize that it would be of benefit to set CDP according to P_L_ and not base it on mean airway pressure (Paw_mean_).

The following hypotheses were tested:

- Conventional HFOV (HFOV_con_) has a negative effect on cardiac function and hemodynamics at higher CDP levels
- There is a difference between the hemodynamic effects of conventional HFOV_conv_ and HFOV guided by transpulmonary pressures (HFOV P_Lmean_)
- HFOV P_Lmean_ not only reduces cardiac depression, but also causes less pulmonary overdistention
- HFOV P_Lmean_ increases non-ventilated lung areas and will therefore worsen gas exchange

## Methods

The study had the approval of our institution’s animal study review board. The animals were handled according to the Helsinki convention for the use and care of animals.

### Animal preparation

Eight healthy pigs (Göttinger mini-pigs, mean weight 41.7 ± 4.0 kg) were premedicated with 40 mg azaperonium intramuscular (i.m.). After cannulating an ear vein, anesthesia was induced with propofol (2 mg kg^− 1^ intravenous (i.v.)) and fentanyl (0.2 μg i.v.), and maintained with infusions of ketamine (10 mg kg^− 1^ h^− 1^) and midazolam (1 mg kg^− 1^ h^− 1^). Ringer acetate was infused at an average rate of 4–5 ml kg^− 1^ h^− 1^.

A cuffed tracheal tube was inserted and the lungs were ventilated in VCV mode (PEEP 5 cmH_2_O; inspiration: expiration ratio (I:E) = 1:1.5; fraction of inspired oxygen (FiO_2_) = 1.0; respiratory rate 15 min^− 1^; constant inspiratory flow; tidal volume V_T_ = 6 ml kg^− 1^). The respiratory rate was adjusted to maintain normocapnia with a maximum rate of 20 min^− 1^. End-tidal CO_2_ (Datex Capnomac Ultima®, Finland), peripheral oxygen saturation, electrocardiogram (ECG) and non-invasive blood pressure were monitored continuously (Datex – Ohmeda S/3 patient monitor, GE, USA).

A thermistor-tipped fiberoptic catheter (Pulsiocath®, 4F FT PV 2024, Pulsion Medical System, Munich, Germany) was placed in a femoral artery. A pulmonary artery catheter (Volef®, Pulsion Medical System, Munich, Germany) was inserted through an 8.5 French sheath introducer in the right internal jugular vein, and the position of the catheter tip was confirmed by pressure tracing. The catheters were connected to pressure transducers and to an integrated bedside monitor (PiCCO®, Volef, Pulsion Medical Systems).

An esophageal balloon catheter (AVEA ®, Care Fusion, Yorba Linda, CA, USA) was inserted to measure esophageal pressure. The correct placement of the catheter was confirmed as described by Talmor et al. [12].

### Experimental protocol

Baseline measurements were performed at 5 cmH_2_O PEEP after all parameters had been constant for 30 min, first in healthy lungs and then after ARDS had been induced by the intrabronchial installation of hydrochloric acid (0.1 N, pH 1.1; 2.5 ml kg^− 1^ body weight) during inspiration. Equal aliquots were instilled through a suction catheter into the right and left main bronchus. The injury was considered stable if partial pressure of arterial oxygen (PaO_2_) remained constantly lower than 300 mmHg at a FiO_2_ of 1.0 at 60 min after instillation.

**Fig. 1:**
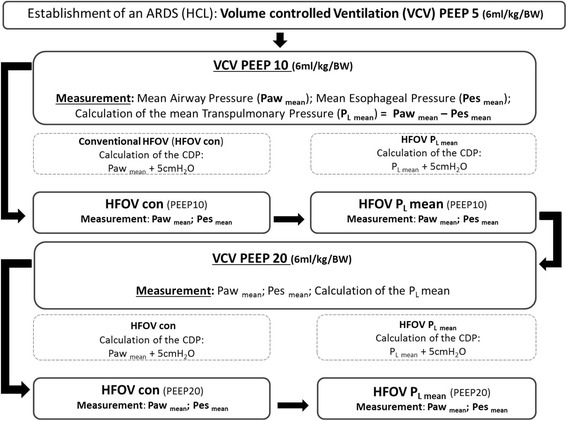
Experimental procedure. ARDS, acute respiratory distress syndrome; HCL, hydrochloric acid; PEEP, positive end-expiratory pressure; BW, body weight; Paw mean, mean airway pressure; Pes mean, mean esophageal pressure; P_L_, transpulmonary pressure; CDP, continuous distending pressure; HFOVcon, conventional high frequency oscillatory ventilation group; HFOV P_L_, transpulmonary guided high frequency oscillatory ventilation group. Significant *P* value (P-Level) <0.05

The animals were then ventilated in the study modes at consecutive PEEP levels of 10 and 20 cmH_2_O. Measurements were performed after 10 min ventilation at each PEEP level. Mean airway (Paw_mean_) and esophageal pressures (Pes) were recorded. End-expiratory esophageal pressure was measured during an end-expiratory hold (PEEP) and the inspiratory esophageal pressure was measured during an inspiratory hold (plateau pressure). The end-expiratory and the end-inspiratory esophageal pressure were then added and divided through the arithmetic mean, in order to calculate the mean esophageal pressure (Pes_mean_). The transpulmonary pressures (P_L_) were then calculated (Fig. 1):

$$ {\mathsf{P}}_{\mathsf{L}\ \mathsf{mean}}={\mathsf{P}\mathsf{aw}}_{\mathsf{mean}}-{\mathsf{P}\mathsf{es}}_{\mathsf{mean}}. $$

At the end of the measurements at each PEEP level the lungs were allowed to collapse by disconnecting the tracheal tube from the respirator for 30 s. A recruitment maneuver was then performed by inflating the lungs to a pressure of 40 cmH_2_O for 40 s after which ventilation was started at the next PEEP level.

VCV was performed as described above. HFOV was performed with a SensorMedics®-Ventilator 3100B (Care Fusion, Yorba Linda, CA, USA). For HFOV_con_ the CDP was set at 5 cmH_2_O above the Paw_mean_. For HFOV P_Lmean_ the CDP was set at 5 cmH_2_O over the mean P_L_ measured during VCV at the corresponding PEEP level as described by Talmor et al. [12] (Fig. 1). The initial ventilator settings were bias flow 20 l min^− 1^, power 70%, inspiration time 44%, and frequency 5 Hz. It was not possible to randomize the order of these measurements due to the nature of the study design.

### Lung imaging and analysis

Computed tomography (CT) scans of the lungs were obtained from apex to base during an end-expiratory hold at a PEEP of 5 cmH_2_O (GE Light Speed VCT, GE Medical Systems, thickness 5 mm, interval 0.5 mm, 100 mA, 100 kV). The method used for quantitative image analysis has been described previously [14]. Quantitative analysis of the entire lung was performed to assess lung density (Hounsfield units, HU), total lung volume, and extent of lung tissue aeration (none, poor, normal, or over-aerated).

Pulmonary parenchyma with a CT density ranging from − 1000 to − 900 HU was classified as overinflated, − 900 to − 500 HU as normal, − 500 to − 100 HU as poorly aerated, and − 100 to + 300 HU as non-aerated (atelectatic).

### Measurements

Cardiac output (CO), stroke volume, right end-diastolic volumes, pulmonary artery pressures, central venous pressures, extravascular lung water index (ELWI), and intrathoracic blood volume index (ITBI) were measured. Cardiac output measurements were performed in triplicate by the same investigator using bolus injections of 20 ml ice-cold 0.9% saline. Arterial samples were collected and blood gases were analyzed immediately (ABL 510, Radiometer, Copenhagen, Denmark).

### Data acquisition

Data recording and analysis was performed using the Modular Intensive Care Data Acquisition System (MIDAS) developed by P. Herrmann and P. Nguyen (Institut für Biomedizinische Technik, Hochschule Mannheim, Germany).

### Statistical analysis

The data were analyzed and the figures created with the statistical software R (www.r-project.org). Data are presented as median and interquartile range (IQR). Changes from baseline in each individual series were assessed using the Wilcoxon test for paired samples.

## Results

### Lung

#### Gas exchange and continuous distending pressures (CDP)

**Fig. 2:**
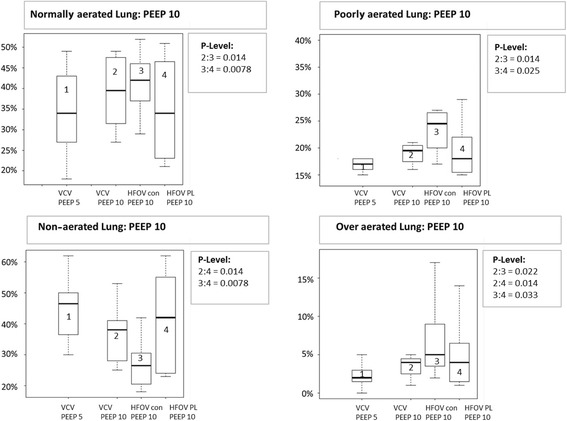
Normally aerated, poorly aerated, non-aerated, and over aerated lung tissue at positive end-expiratory pressure (PEEP) 10. Data are presented as median, 25th and 75th quartiles, and minimum and maximum (n = 8). VCV, volume controlled ventilation; HFOV_con_, conventional high frequency oscillatory ventilation group; HFOV P_L_, mean transpulmonary pressure guided high frequency oscillatory ventilation group. Box plots are numbered from the left to the right side from 1 to 4. Significant *P* value (P-Level) <0.05

PaO_2_ decreased and paCO_2_ increased after induction of ARDS. PaCO_2_ was significantly lower in both HFOV groups than in the volume-controlled ventilation groups (VCV), except at a PEEP level of 10 cm H_2_0 in the transpulmonary pressure (P_L_)-guided group (Table 1). There was no difference in paO_2_ between HFOV_con_ and HFOV P_Lmean_ at any PEEP level. The CDP based on mean P_L_ was approximately 40% lower than that based on mean airway pressures (Fig. 2).

**Table 1:** Pulmonary gas exchange, serum lactate and airway pressures

	T_0_ PEEP 5			ARDS PEEP 5			ARDS PEEP 10			ARDS PEEP 20		
pHa	Median	25%	75%	Median	25%	75%	Median	25%	75%	Median	25%	75%
VCV	7.47	7.46	7.50	7.36	7.35	7.39	7.34^	7.32	7.42	7.31	7.28	7.34
ARDS HFOV _con_	–	–	–	–	–	–	7.54	7.49	7.58	7.51	7.47	7.52
ARDS HFOV P_L mean_	–	–	–	–	–	–	7.46	7.36	7.52	7.58	7.51	7.59
PaCO_2_, mmHg												
VCV	41.5	38.3	42.8	45.0^#^	43.0	46.5	50.5^	46.3	52.0	51.0©	48.3	55.3
ARDS HFOV _con_	–	–	–	–	–	–	28.0 ^◯^	27.0	30.3	29.5	27.0	33.3
ARDS HFOV P_L mean_	–	–	–	–	–	–	40.5	32.8	47.5	28.0	25	29.3
PaO_2_, mmHg												
VCV	666.0*	647.8	675.8	71.0	65.3	80.8	54.0	50.8	71.8	88.0	46.0	121.8
ARDS HFOV _con_	–	–	–	–	–	–	51.0	39.8	71.8	67.5	39.0	105.8
ARDS HFOV P_L mean_	–	–	–	–	–	–	43.5	40.5	52.8	63.0	53.0	131.1
Lactate, mmol/l												
VCV	1.9*	1.5	2.4	2.4	1.6	3.1	2.4	2.0	2.8	2.8	2.4	3.3
ARDS HFOV _con_	–	–	–	–	–	–	2.7	2.5	3.2	2.6	2.5	3.0
ARDS HFOV P_L mean_	–	–	–	–	–	–	2.6	2.1	3.0	2.8	2.6	3.4
Airway pressures												
Plateau airway pressure VCV	14.5	14.0	16.0	24.5	23.8	26.0	29.5	28.0	30.3	38.5	37.8	39.0
Mean air pressure												
VCV	8.0	8.0	8.7	11.5	11.3	12.0	16.5	16.0	16.8	26.1	25.9	26.3
HFOV _con_	–	–	–	–	–	–	20.5	19.5	21.0	30.0	30.0	30.0
HFOV P_L mean_	–	–	–	–	–	–	11.0	11.0	16.3	20.5	17.0	22.8
Mean esophageal pressure												
VCV	4.5	1.2	8.4	4.5	3.7	9.2	10.0	7.1	12.3	12.2	10.2	14.3
HFOV _con_	–	–	–	–	–	–	10.0	7.1	12.3	11.5	9.3	14.3
HFOV P_L mean_	–	–	–	–	–	–	8.5	7.0	9.3	9.0	7.0	11.5
P_L mean_												
VCV	3.8	0.0	6.8	6.7	3.4	7.3	6.3	4.1	9.7	13.5	11.9	15.5
HFOV _con_	–	–	–	–	–	–	9.5	6.5	13.3	18.5	15.8	20.8
HFOV P_L mean_	–	–	–	–	–	–	3.0	1.5	9.0	11.5	7.8	15.8

Values are medians (25th and 75th quartiles) in eight animals

*T*_*0*_ *PEEP 5* start of the experiment without acute respiratory distress syndrome (ARDS) and positive end-expiratory pressure (PEEP = 5 cmH_2_O), *ARDS PEEP 5/10/20* ARDS with PEEP of 5, 10, and 20 cm H_2_O, *VCV* conventional volume controlled ventilation, *HFOV* high frequency oscillatory ventilation, *HFOV con* conventional high frequency oscillatory ventilation, *HFOV P*_*L*_ mean HFOV guided by mean transpulmonary pressure, *pHa* pH in arterial blood, *PaCO*_*2*_ arterial carbon dioxide tension, *PaO*_*2*_ arterial oxygen tension, *Paw* mean airway pressure, *P*_*L*_ transpulmonary pressure (P_L_ = Paw-esophageal pressure)

*p < 0.05 VCV T_0_ PEEP 5 vs. VCV ARDS PEEP 5; ^*p* < 0.05 VCV ARDS PEEP 10 vs. HFOV con “PEEP 10”; ^♀^*p* < 0.05 VCV ARDS PEEP 10 vs. HFOV P_L_ mean “PEEP 10”; ^◯^*p* < 0.05 HFOV con ARDS “PEEP 10” vs. HFOV P_L_ mean ARDS “PEEP 10”; ©*p* < 0.05 VCV ARDS PEEP 20 vs. HFOV con “PEEP 20”; ^£^*p* < 0.05 VCV ARDS PEEP 20 vs. HFOV P_L_ mean “PEEP 20”; ^Ω^*p* < 0.05 HFOV con ARDS “PEEP 20” vs. HFOV P_L_ mean ARDS “PEEP 20”; *p* < 0.05 (*p* values were determined using the Wilcoxon test for paired samples)

#### Lung density and total lung volume and aeration

**Fig. 3:**
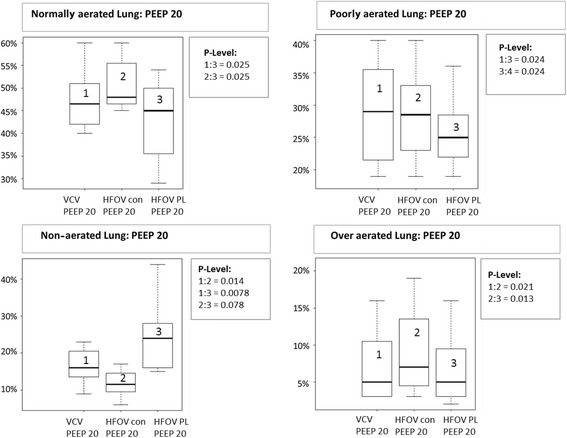
Normally aerated, poorly aerated, non-aerated and over aerated lung tissue at positive end-expiratory pressure (PEEP) 20. Data are presented as median, 25th and 75th quartiles, and minimum and maximum (n = 8). VCV, volume controlled ventilation; HFOV_con_, conventional high frequency oscillatory ventilation group; HFOV P_L_, mean transpulmonary pressure guided high frequency oscillatory ventilation group. Box plots are numbered from the left to the right side from 1 to 3. Significant *P* value (P-Level) <0.05

Total lung density expressed in mean HU, total lung volume and percentage of normally, poorly, non-aerated and over-aerated lung tissue is shown in Table 2. Lung density increased significantly during HFOV P_Lmean_ while it stayed the same during HFOV_con_ compared to VCV at PEEP 10 (*p* < 0.05) (Fig. 3). Furthermore there was a significant increase in density during HFOV P_Lmean_ compared to HFOV_con_ at PEEP 10. At PEEP 20, lung density decreased during HFOV_con_ and increased during HFOV P_Lmean_ compared to VCV. There was also a significant difference in lung density between HFOV_con_ and HFOV P_Lmean_ (*p* < 0.05) (Fig. 3).

**Table 2:** Lung density, total lung volume, normally aerated, poorly aerated, non-aerated and over aerated lung tissue

		ARDS PEEP 5			ARDS PEEP 10			ARDS PEEP 20		
Hounsfield units	HU	Median	25%	75%	Median	25%	75%	Median	25%	75%
VCV expiration		− 313.6	− 379.0	− 266.0	−379.2^♀^	− 452.6	− 331.1	− 479.5©^£^	− 554.7	− 439.5
ARDS HFOV _con_		–	–	–	− 418.1^◯^	−517.5	− 398.9	− 535.4 ^Ω^	− 580.3	− 484.6
ARDS HFOV P_L mean_		–	–	–	−338.6	− 477.2	− 238.9	− 447.1	− 549.4	− 399.0
Total lung volume	ml									
VCV		1351.6	1197.8	1480.9	1619.3^	1553.5	1765.6	2349.2©	2230.8	2435.9
ARDS HFOV _con_		–	–	–	1976.6^◯^	1870.1	2249.7	2582.1 ^Ω^	2468.3	2734.3
ARDS HFOV P_L mean_		–	–	–	1817.4	1464.8	1955.6	2256.0	2159.2	2388.3
Normally aerated tissue	ml									
VCV expiration		484.3	387.5	509.9	647.9^♀^	591.8	725.6	1112.9^£^	1016.3	1157.6
ARDS HFOV _con_		–	–	–	855.4^◯^	812.7	937.7	1292.6 ^Ω^	1196.9	1424.4
ARDS HFOV P_L mean_		–	–	–	593.3	418.5	881.7	979.0	885.3	1166.5
Poorly aerated tissue	ml									
VCV expiration		225.1	217.2	246.3	306.1^	283.7	341.6	621.6^£^	509.5	818.3
ARDS HFOV _con_		–	–	–	447.9^◯^	390.5	507.2	685.9 ^Ω^	596.9	776.6
ARDS HFOV P_L mean_		–	–	–	336.1	221.4	413.1	557.2	500.1	614.5
Non-aerated tissue	ml									
VCV expiration		628.3	447.0	725.3	608.4^♀^	448.8	705.6	409.3©^£^	325.5	459.0
ARDS HFOV _con_		–	–	–	567.9^◯^	450.5	610.9	350.2 ^Ω^	250.6	367.5
ARDS HFOV P_L mean_		–	–	–	736.5	484.1	775.7	518.6	391.6	642.4
Over aerated tissue	ml									
VCV expiration		26.3	19.3	32.2	59.5^^♀^	47.2	68.0	124.8©	71.1	218.0
ARDS HFOV _con_		–	–	–	106.1^◯^	72.1	167.7	180.7 ^Ω^	113.7	390.7
ARDS HFOV P_L mean_		–	–	–	72.9	23.6	107.4	121.0	63.6	208.4

Values are medians (25th and 75th quartiles) in eight animals. See text or Table 1 for description of groups

*ARDS* acute respiratory distress syndrome, *VCV* volume controlled ventilation, *HFOV con* conventional high frequency oscillatory ventilation, *HFOV P*_*L*_ mean HFOV guided by mean transpulmonary pressure, *PEEP* positive end-expiratory pressure

^*p* < 0.05 VCV ARDS PEEP 10 expiration vs. HFOV con “PEEP 10”; ^♀^*p* < 0.05 VCV ARDS PEEP 10 expiration vs. HFOV P_L_ mean “PEEP 10”; ^◯^*p* < 0.05 HFOV con ARDS “PEEP 10” vs. HFOV P_L_ mean ARDS “PEEP 10”; ©*p* < 0.05 VCV ARDS PEEP 20 expiration vs. HFOV con “PEEP 20”; ^£^*p* < 0.05 VCV ARDS PEEP 20 expiration vs. HFOV P_L_ mean “PEEP 20”; ^Ω^*p* < 0.05 HFOV con ARDS “PEEP 20” vs. HFOV P_L_ mean ARDS “PEEP 20”; *p* < 0.05 (*p* values were determined using the Wilcoxon test for paired samples)

**Fig. 4:**
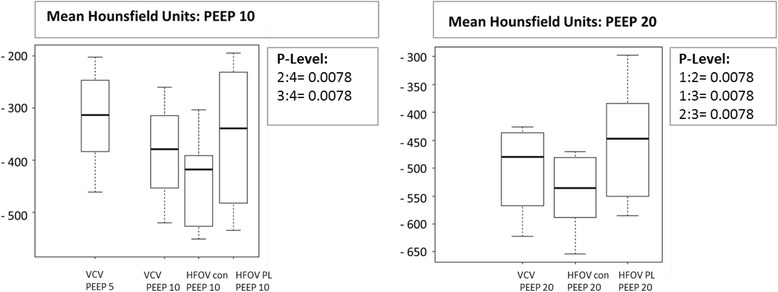
Mean Hounsfield units at positive end-expiratory pressure (PEEP) 10 and 20. Data are presented as median, 25th and 75th quartiles, and minimum and maximum (n = 8). VCV, volume controlled ventilation; HFOV_con_, conventional high frequency oscillatory ventilation group; HFOV P_L_, mean transpulmonary pressure guided high frequency oscillatory ventilation group. Box plots are numbered from the left to the right side from 1 to 3 and 1 to 4. Significant *P* value (P-Level) <0.05

**Fig. 5:**
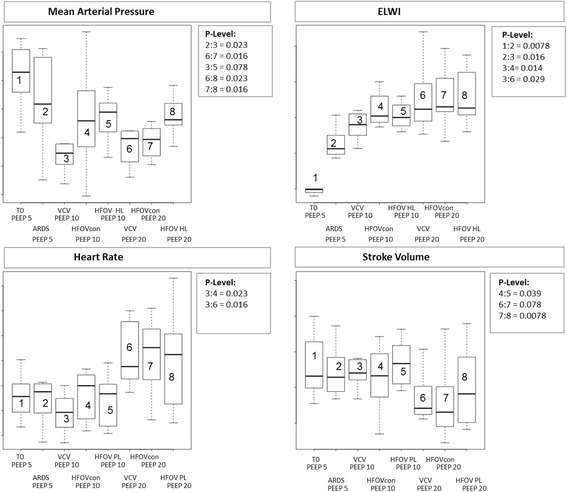
Mean arterial pressure, extra vascular lung water index (ELWI), heart rate and stroke volume. Data are presented as median, 25th and 75th quartiles, and minimum and maximum (n = 8). T0, start of the measurement process; ARDS, established acute respiratory distress syndrome; VCV, volume controlled ventilation; HFOV_con_, conventional high frequency oscillatory ventilation group; HFOV P_L_, mean transpulmonary pressure guided high frequency oscillatory ventilation group. Box plots are counted from the left to the right side from 1 to 8. Significant *P* value (P-Level) <0.05

Total lung volume was greater with HFOV_con_ than with HFOV P_Lmean_. Roughly summarized, there was significantly more normally and poorly aerated lung tissue with HFOV_con_, while less over-aerated and more non-aerated lung tissue was observed with HFOV P_Lmean_ (Figs. 4 and 5).

#### Extravascular lung water

The extravascular lung water index (ELWI) increased after induction of ARDS (*p* < 0.05), but there was no difference between HFOV P_Lmean_ and HFOV_con_.

#### Hemodynamics and cardiac function

Heart rate (HR), MAP, central venous pressue (CVP), mean pulmonary arterial pressure (mPAP), CO, stroke volume (SV), intrathoracic blood volume index (ITBI) and ELWI are shown in Table 3. Mean PAP, right ventricular end-diastolic volume index (RVEDI), and ELWI increased significantly after induction of ARDS.

**Table 3:** Hemodynamic parameters

	T_0_ PEEP 5			ARDS PEEP 5			ARDS PEEP 10			ARDS PEEP 20		
HR, min^− 1^	Median	25%	75%	Median	25%	75%	Median	25%	75%	Median	25%	75%
VCV	55.7	50.2	59.9	57.6	49.7	60.4	49.3^#^^	44.6	53.9	67.6	63.6	84.2
ARDS HFOV _con_	–	–	–	–	–	–	60.0	48.5	63.8	75.2	66.1	79.5
ARDS HFOV P_L mean_	–	–	–	–	–	–	56.7	43.9	60.5	72.5	54.4	77.4
MAP, mmHg												
VCV	82.9	76.2	89.2	71.8	65.4	86.7	54.6^#♀^	50.9	57.7	59.7©^£^	52.5	62.3
ARDS HFOV _con_	–	–	–	–	–	–	65.9	57.3	74.6	59.4 ^Ω^	54.4	62.8
ARDS HFOV P_L mean_	–	–	–	–	–	–	68.9	62.2	70.7	66.3	64.6	70.8
CVP, mmHg												
VCV	12.5	11.0	14.5	11.5	8.5	12.3	7.0^#^^^♀^	6.0	9.5	10.0	7.8	11.5
ARDS HFOV _con_	–	–	–	–	–	–	9.5	7.8	10.5	10.5	9.0	12.3
ARDS HFOV P_L mean_	–	–	–	–	–	–	8.0	7.0	10.5	10.0	8.0	12.3
mPAP, mmHg												
VCV	19.3*	17.6	21.3	25.4	23.3	28.1	22.1^#^	18.7	25.1	26.6	21.8	27.7
ARDS HFOV _con_	–	–	–	–	–	–	21.9	19.1	31.3	25.6	21.2	31.4
ARDS HFOV P_L mean_	–	–	–	–	–	–	21.2	17.1	26.1	24.0	21.7	26.7
CO, l.min^−1^												
VCV	2.2	2.1	2.5	2.6	2.4	3.1	2.4	2.2	2.6	2.4©	2.1	2.6
ARDS HFOV _con_	–	–	–	–	–	–	2.5	2.3	2.8	2.1 ^Ω^	1.8	2.5
ARDS HFOV P_L mean_	–	–	–	–	–	–	2.4	2.2	3.0	2.5	2.2	2.9
SV, ml												
VCV	46.4	41.1	63.9	45.7	38.2	55.2	48.1^#^	45.0	55.3	28.3©	25.5	39.9
ARDS HFOV _con_	–	–	–	–	–	–	46.5^◯^	38.0	58.4	26.1 ^Ω^	18.9	37.2
ARDS HFOV P_L mean_	–	–	–	–	–	–	53.4	42.2	59.0	36.4	20.9	54.2
RVEDI, ml m^−2^												
VCV	90.3*	86.2	97.5	104.0	97.7	127.6	103.9	96.1	110.5	102.2	90.6	120.6
ARDS HFOV _con_	–	–	–	–	–	–	102.8	101.1	115.9	86.6	71.3	101.3
ARDS HFOV P_L mean_	–	–	–	–	–	–	119.5	99.3	133.6	98.5	94.1	100.4
ITBI; ml m^2^												
VCV	550.0	514.4	603.1	637.2	586.5	718.8	638.5	604.8	708.9	575.8©^£^	513.5	651.8
ARDS HFOV _con_	–	–	–	–	–	–	651.7	611.3	706.1	560.7 ^Ω^	506.8	665.9
ARDS HFOV P_L mean_	–	–	–	–	–	–	660.6	639.6	733.0	621.0	573.0	675.7
ELWI, ml kg^−1^												
VCV	4.9*	4.6	5.0	10.6	9.9	12.4	14.0^#^^	12.8	15.3	16.2	14.5	19.1
ARDS HFOV _con_	–	–	–	–	–	–	15.2	14.3	17.0	16.5	15.9	20.3
ARDS HFOV P_L mean_	–	–	–	–	–	–	15.0	14.1	16.1	16.3	15.7	20.5

Values are medians (25th and 75th quartiles) in eight animals. See text or Table 1 for description of groups

*HR* heart rate, *MAP* mean arterial pressure, *CVP* central venous pressure, *mPAP* mean pulmonary artery pressure, *PCWP* pulmonary capillary wedge pressure, *CO* cardiac output, *SV* stroke volume, *SVV* stroke volume variation, *ITBI* intrathoracic blood volume index, *ELWI* extravascular lung water index, *ARDS* acute respiratory distress syndrome, *PEEP* positive end-expiratory pressure

*p < 0.05 VCV T_0_ PEEP 5 vs. VCV ARDS PEEP 5; ^#^*p* < 0.05 VCV ARDS PEEP 10 vs. VCV ARDS PEEP 20; ^*p* < 0.05 VCV ARDS PEEP 10 vs. HFOV con “PEEP 10”; ^♀^*p* < 0.05 VCV ARDS PEEP 10 vs. HFOV P_L_ mean “PEEP 10”; ^◯^*p* < 0.05 HFOV con ARDS “PEEP 10” vs. HFOV P_L_ mean ARDS “PEEP 10”; ©*p* < 0.05 VCV ARDS PEEP 20 vs. HFOV con “PEEP 20”; ^£^*p* < 0.05 VCV ARDS PEEP 20 vs. HFOV P_L_ mean “PEEP 20”; ^Ω^*p* < 0.05 HFOV con ARDS “PEEP 20” vs. HFOV P_L_ mean ARDS “PEEP 20”; *p* < 0.05 (*p* values were determined using the Wilcoxon test for paired samples)

**Fig. 6:**
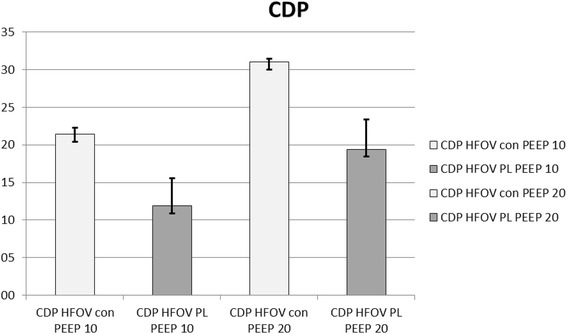
Comparison of the continuous distending airway pressures (CDP) guided by the mean airway pressure (Paw mean) and the mean transpulmonary pressure (P_L mean_). Data are presented as mean and standard deviation (n = 8). CDP, continuous distending pressure; CDP Paw mean HFOV_con_, conventional high frequency oscillatory ventilation group; HFOV P_L_, mean transpulmonary pressure guided high frequency oscillatory ventilation group

During volume-controlled ventilation, HR, CVP, mPAP, MAP, and ELWI increased after the change from PEEP 10 to PEEP 20, while SV decreased (*p* < 0.05). SV was larger during HFOV P_Lmean_ than during HFOV_con_ at PEEP 10. At PEEP 20, SV and MAP, CO, and ITBI were greater during HFOVP_Lmean_ than during HFOV_con_ (*p* < 0.05) (Table 3; Fig. 6).

## Discussion

To our knowledge this is the first study in animals that compares the effects of two HFOV regimens on systemic hemodynamics, gas exchange, and lung aeration; one in which the continuous distending pressure (CDP) was adjusted according to mean airway pressure (HFOV_con_), and one adjusted to the corresponding mean transpulmonary pressure (HFOV P_Lmean_).

The main finding of the present study is that transpulmonary pressure-guided HFOV with high PEEP values has less impact on systemic hemodynamics than conventional HFOV and does not compromise oxygenation. The reduction in distending pressures (CDP) associated with transpulmonary pressure-guided HFOV resulted in less pulmonary overdistension, but increased the percentage of non-aerated lung tissue (Figs. 3, 4 and 5). Furthermore on comparison between VCV and transpulmonary pressure-guided HFOV there was higher MAP and ITBI and a lower percentage of normal and poor ventilated lung tissue, but less pulmonary overdistension at high PEEP levels in HFOV P_Lmean_.

In previous studies of conventional HFOV, the CDP was based on the mean airway pressure at each PEEP level [4, 7, 8, 11, 15]. This universally established procedure of setting CDP as airway pressure + 5cmH_2_0 is merely an empirical convention that is not underpinned by experimental evidence. It is known that one cannot equate mean airway pressure and transpulmonary pressure, particularly not in patients with ARDS, because of the changes in chest wall and lung elastance. Using Paw or plateau pressure as the reference point would most likely yield a CDP that was too high and could cause overdistension of the lung and, in the end, ventilator-induced lung injury (VILI).

For the sake of comparison in the present study, CDP was set at 5 cmH_2_O above the mean transpulmonary pressure at each corresponding PEEP level. This is also an empirical approach, albeit it an approach that induces only one modification and not the additional factor of a different pressure increment over the reference point.

Talmor et al. [12] have already shown that H_L_-guided ventilation is superior to conventional mechanical ventilation. In this study the CDP levels based on P_Lmean_ were approximately 40% than those based on mean airway pressures at both employed PEEP levels.

The lesser degree of adverse circulatory effects compared to those observed in the conventionally ventilated animals or described in recently published studies on HFOV is possibly due to the lower CDP used in HFOV P_Lmean_ [15, 16]. These circulatory effects are probably caused by an intrathoracic pressure-related preload reduction or by direct impairment of right ventricular function [15, 17]. Most HFOV studies in the past did not take the hemodynamic instability of patients with ARDS into account, which was the consequence of the strict fluid reduction in ARDS therapy [18]. HFOV employed under conditions of hypovolemia will reduce pulmonary perfusion and affect oxygenation. This was confirmed in a study by Ursulet et al. [19], who showed that HFOV indeed caused a significant reduction in cardiac index, but not in arterial blood pressure in hypovolemic patients. Echocardiography or hemodynamic evaluation should therefore be performed before HFOV is started in order to reduce the potential negative circulatory effects. An animal study by Songqiao and coworkers [20] demonstrated that almost no hemodynamic depression actually occurs if the CDP is carefully titrated.

The lower CDP in our study resulted in a higher percentage of non-aerated lung tissue because the higher distending pressures in conventional HFOV are comparable to high PEEP levels. High PEEP levels and a correspondingly high CDP can recruit lung tissue but on the other hand it can also lead to lung overdistension [21]. Fu et al. showed that lung overdistension triggered by an increase in transpulmonary pressure produced a significant increase in the number of epithelial and endothelial breaks [22], which can cause pulmonary edema. Parker et al. are confident that microvascular permeability might be actively modulated by a cellular response due to overdistension [23]. The authors assumed that this cellular response might be initiated by stretch-activated cation channels. The 3.7-fold increase in the capillary filtration coefficient found in their study is a strong argument for avoiding overdistension. It is noteworthy that there was no difference in oxygenation between the two groups, although the animals in the P_Lmean_ group had a greater percentage of non-ventilated lung tissue. This might be explained by the fact that the young animals had a more robust hypoxic pulmonary vasoconstriction (HPV) reflex [24] so that perfusion was reduced in the lung areas that were no longer ventilated. The situation in patients in intensive care might be a different one.

Not only overdistension, but also high oxygen concentrations can cause lung injury. HFOV initiated late in the course of ARDS will require a high FiO_2_, and high oxygen concentrations in combination with low distending pressures tend to promote airway closure with consequent atelectasis in dependent regions [25]. Derosa et al. showed in a porcine model of ARDS that no alveolar collapse occurred with low FiO_2_ and low distending pressures. One can therefore safely conclude that the FiO_2_ of 1.0 in our study increased the amount of non-ventilated lung tissue. High distending pressures can prevent lung collapse but they also cause the cyclical alveolar opening and closing that increases lung injury. HFOV should therefore not be simply regarded as a rescue therapy but rather as an early therapeutic option, because in the early stage of ARDS a low FiO_2_ and low distending pressures will be sufficient therapy.

Although spontaneous ventilation is a cornerstone of ARDS therapy, muscle relaxation in the early phase can reduce lung injury [26]. Muscle relaxation facilitates ventilator synchronization and thus helps to limit alveolar pressure peaks with overdistension and consecutive pulmonary or systemic inflammation [26]. But it also increases the percentage of non-ventilated tissue. In view of our results, transpulmonary pressure-guided HFOV probably has a similar effect because it reduces overdistension. The results of the OSCILLATE and the OSCAR trials called the safety of HFOV into question [9, 10]. The OSCILLATE trial was terminated before completion because the interim analysis had shown that the use of HFOV resulted in a 12% increase in in-hospital mortality. The patients in the HFOV group had required more vasopressor support, perhaps due to the high intrathoracic pressures used in the OSCILLATE trial. High intrathoracic pressures cause hemodynamic compromise and increased right ventricular afterload. Employing transpulmonary pressure-guided HFOV would have resulted in lower mean airway pressures and hemodynamic compromise would have been less severe. It is also important to select suitable patients because HFOV is probably only a superior method in patients with homogenously damaged lungs [27], which are potentially recruitable for gas exchange. It should also be emphasized that centers with little or no experience in the use of HFOV participated in both trials, so the question arises whether suitable patients had been selected, and if HFOV had been correctly implemented.

The high airway pressures used in conventional ventilation or conventional HFOV induce regional overdistension in healthy lung units, which is probably the reason why the open-lung concept has failed to reduce mortality in ARDS in the past. One should note that the OSCAR trial, in which there was no difference in mortality between HFOV and conventional ventilation, used lower airway pressures than the OSCILLATE trial. Overdistension, and to some degree even recruitment, causes local and systemic inflammation, which leads to the question whether a larger percentage of non-aerated lung tissue, as found in our study, might actually be an advantage. It should be noted that on comparison between VCV and HFOV P_Lmean_ there were fewer differences in hemodynamics than on comparison between HFOV_con_ and HFOV P_Lmean_. Only the MAP and the ITBI were higher in HFOV P_Lmean_ compared to VCV, but SV and CO stayed the same at high PEEP levels in comparison to HFOV_con_ and HFOV P_Lmean_. CT examinations of HFOV P_Lmean_ and VCV were comparable to HFOV P_Lmean_ versus HFOV_con_, because a higher percentage of non ventilated and poorly ventilated lung tissue was observed, but there was less over distended lung tissue in HFOV P_Lmean_.

We propose that HFOV guided by transpulmonary pressure monitoring can be an alternative therapeutic option in the early stage of ARDS because it reduces the amount of overdistension and thereby limits escalation of lung injury.

### Limitations

The primary limitation of the study was that it was not possible to randomize the order in which the ventilatory modes were applied, since the transpulmonary pressures used for the HFOV settings were determined during the preceding phase with conventional ventilation. There is the possibility, albeit a small one, that using each animal for both ventilator modes might have induced factors relating to the history of the lung, which as a consequence might have influenced subsequent measurements. However, performing all measurements in a single animal has the major advantage of reducing inter-individual variability and allows the use of paired-data analysis that gives greater statistical power and reduces the risk of type II error. Statistical analysis was exploratory and differences in median and interquartile ranges were reported. Significance was assessed using the paired Wilcoxon test, but was not adjusted for multiple testing in order to avoid false negatives.

Another limitation is the fact that the hemodynamic advantages of HFOV P_Lmean_ over HFOV_con_ were only detectable at a very high PEEP level of 20 cmH_2_O. The plateau pressures of more than 30 cmH_2_O associated with this PEEP level would not have been tolerated in a clinical setting. The lower, clinically acceptable Paw would have resulted in a lower CDP during HFOV_con_ and there might have been no difference detectable at this pressure.

Last, the CDP used for HFOV P_Lmean_ was obtained by a method analogous to that used for HFOV_con_, i.e. by adding 5 cmH_2_O to the reference pressure, in this case P_L_. This is also an empirical approach and has no experimental basis.

## Conclusions

When treating ARDS, the ventilator settings demand meticulous adjustments and are a compromise between recruiting and stabilizing non-aerated lung tissue while avoiding overdistention and hemodynamic compromise. Our study results showed that HFOV guided by transpulmonary pressure is equal or superior to conventional HFOV with regard to systemic hemodynamics, oxygenation, and lung overdistension in animals. It might therefore be useful as a prophylactic approach to prevent worsening of lung injury in the early phase of ARDS. The promising results of transpulmonary pressure-guided HFOV would justify a clinical trial in which HFOV is initiated immediately after the onset of ARDS.

## Abbreviations

ARDS: Acute respiratory distress syndrome
BW: Body weight
CDP: Continuous distending pressure
cmH_2_O: Centimeter of water
CO: Cardiac output
CO_2_: Carbon dioxide
CT: Computer tomography
CVP: Central venous pressure
ELWI: Extravascular water index
F: French
FiO_2_: Fraction of inspired oxygen
HCl: Hydrochloric acid
HFOV P_L mean_: High frequency oscillatory ventilation; mean transpulmonary pressure-guided
HFOV: High frequency oscillatory ventilation
HFOV_con_: High frequency oscillatory ventilation; conventional mode
HR: Heart rate
HU: Hounsfield units
i.v.: Intravenous
I:E: Inspiratory: expiratory ratio (I: inspiration; E: expiration)
ITBI: Intrathoracic blood volume index
Kg: Kilogram
MAP: Mean arterial pressure
mg: Milligram
MIDAS: Modular Intensive Care Data Acquisition System
ml: Milliliter
mPAP: Mean pulmonary arterial pressure
n: Number
p: P level
PaO_2_: Partial arterial oxygen pressure
PaCO_2_: Partial arterial CO_2_ pressure
Paw mean: Mean airway pressure
PEEP: Positive end-expiratory pressure
Pes _mean_: Mean esophageal pressure
P_L_: Transpulmonary pressure
P_pl_: Pleural pressure
SV: Stroke volume
VCV: Volume controlled ventilation
VT: Tidal volume

## References

[1] Amato MB, Barbas CS, Medeiros DM, Magaldi RB, Schettino GP, Lorenzi-Filho G, Kairalla RA, Deheinzelin D, Munoz C, Oliveira R (1998). Effect of a protective-ventilation strategy on mortality in the acute respiratory distress syndrome. N Engl J Med.

[2] Amato MB, Meade MO, Slutsky AS, Brochard L, Costa EL, Schoenfeld DA, Stewart TE, Briel M, Talmor D, Mercat A (2015). Driving pressure and survival in the acute respiratory distress syndrome. N Engl J Med.

[3] Krishnan JA, Brower RG (2000). High-frequency ventilation for acute lung injury and ARDS. Chest.

[4] Derdak S, Mehta S, Stewart TE, Smith T, Rogers M, Buchman TG, Carlin B, Lowson S, Granton J (2002). High-frequency oscillatory ventilation for acute respiratory distress syndrome in adults: a randomized, controlled trial. Am J Respir Crit Care Med.

[5] Nelle M, Zilow EP, Linderkamp O (1997). Effects of high-frequency oscillatory ventilation on circulation in neonates with pulmonary interstitial emphysema or RDS. Intensive Care Med.

[6] Fort P, Farmer C, Westerman J, Johannigman J, Beninati W, Dolan S, Derdak S (1997). High-frequency oscillatory ventilation for adult respiratory distress syndrome–a pilot study. Crit Care Med.

[7] David M, Weiler N, Heinrichs W, Neumann M, Joost T, Markstaller K, Eberle B (2003). High-frequency oscillatory ventilation in adult acute respiratory distress syndrome. Intensive Care Med.

[8] Mehta S, Lapinsky SE, Hallett DC, Merker D, Groll RJ, Cooper AB, MacDonald RJ, Stewart TE (2001). Prospective trial of high-frequency oscillation in adults with acute respiratory distress syndrome. Crit Care Med.

[9] Young D, Lamb SE, Shah S, MacKenzie I, Tunnicliffe W, Lall R, Rowan K, Cuthbertson BH (2013). High-frequency oscillation for acute respiratory distress syndrome. N Engl J Med.

[10] Ferguson ND, Cook DJ, Guyatt GH, Mehta S, Hand L, Austin P, Zhou Q, Matte A, Walter SD, Lamontagne F (2013). High-frequency oscillation in early acute respiratory distress syndrome. N Engl J Med.

[11] David M, Karmrodt J, Weiler N, Scholz A, Markstaller K, Eberle B (2005). High-frequency oscillatory ventilation in adults with traumatic brain injury and acute respiratory distress syndrome. Acta Anaesthesiol Scand.

[12] Talmor D, Sarge T, Malhotra A, O'Donnell CR, Ritz R, Lisbon A, Novack V, Loring SH (2008). Mechanical ventilation guided by esophageal pressure in acute lung injury. N Engl J Med.

[13] Heuer JF, Sauter P, Barwing J, Herrmann P, Crozier TA, Bleckmann A, Beissbarth T, Moerer O, Quintel M (2012). Effects of high-frequency oscillatory ventilation on systemic and cerebral hemodynamics and tissue oxygenation: an experimental study in pigs. Neurocrit Care.

[14] Rylander C, Tylen U, Rossi-Norrlund R, Herrmann P, Quintel M, Bake B (2005). Uneven distribution of ventilation in acute respiratory distress syndrome. Crit Care.

[15] David M, von Bardeleben RS, Weiler N, Markstaller K, Scholz A, Karmrodt J, Eberle B (2004). Cardiac function and haemodynamics during transition to high-frequency oscillatory ventilation. Eur J Anaesthesiol.

[16] Adhikari NK, Bashir A, Lamontagne F, Mehta S, Ferguson ND, Zhou Q, Hand L, Czarnecka K, Cook DJ, Granton JT (2011). High-frequency oscillation in adults: a utilization review. Crit Care Med.

[17] Guervilly C, Forel JM, Hraiech S, Demory D, Allardet-Servent J, Adda M, Barreau-Baumstark K, Castanier M, Papazian L, Roch A (2012). Right ventricular function during high-frequency oscillatory ventilation in adults with acute respiratory distress syndrome. Crit Care Med.

[18] Roch A, Guervilly C, Papazian L (2011). Fluid management in acute lung injury and ARDS. Ann Intensive Care.

[19] Ursulet L, Roussiaux A, Belcour D, Ferdynus C, Gauzere BA, Vandroux D, Jabot J (2015). Right over left ventricular end-diastolic area relevance to predict hemodynamic intolerance of high-frequency oscillatory ventilation in patients with severe ARDS. Ann Intensive Care.

[20] Liu S, Huang Y, Wang M, Chen Q, Liu L, Xie J, Tan L, Guo F, Yang C, Pan C (2014). Effects of high-frequency oscillatory ventilation and conventional mechanical ventilation on oxygen metabolism and tissue perfusion in sheep models of acute respiratory distress syndrome. Chin Med J.

[21] Briel M, Meade M, Mercat A, Brower RG, Talmor D, Walter SD, Slutsky AS, Pullenayegum E, Zhou Q, Cook D (2010). Higher vs lower positive end-expiratory pressure in patients with acute lung injury and acute respiratory distress syndrome: systematic review and meta-analysis. JAMA.

[22] Fu Z, Costello ML, Tsukimoto K, Prediletto R, Elliott AR, Mathieu-Costello O, West JB (1992). High lung volume increases stress failure in pulmonary capillaries. J Appl Physiol (1985).

[23] Parker JC, Ivey CL, Tucker JA (1998). Gadolinium prevents high airway pressure-induced permeability increases in isolated rat lungs. J Appl Physiol (1985).

[24] Lumb AB, Slinger P (2015). Hypoxic pulmonary vasoconstriction: physiology and anesthetic implications. Anesthesiology.

[25] Derosa S, Borges JB, Segelsjo M, Tannoia A, Pellegrini M, Larsson A, Perchiazzi G, Hedenstierna G (2013). Reabsorption atelectasis in a porcine model of ARDS: regional and temporal effects of airway closure, oxygen, and distending pressure. J Appl Physiol (1985).

[26] Papazian L, Forel JM, Gacouin A, Penot-Ragon C, Perrin G, Loundou A, Jaber S, Arnal JM, Perez D, Seghboyan JM (2010). Neuromuscular blockers in early acute respiratory distress syndrome. N Engl J Med.

[27] Downar J, Mehta S (2006). Bench-to-bedside review: high-frequency oscillatory ventilation in adults with acute respiratory distress syndrome. Crit Care.

